# Quantitative assessment of lower eyelid morphology following orbital floor fracture repair

**DOI:** 10.1016/j.jpra.2026.09.009

**Published:** 2026-09-15

**Authors:** Miki Nakanishi, Hikaru Watanabe, Suzuna Ishimoto, Takuya Suzuki, Toshihiko Nishioka, Kazuhisa Uemura, Yasuhiro Sakata, Shinichi Asamura

**Affiliations:** a Department of Plastic and Reconstructive Surgery, Kishiwada Tokushukai Hospital, Kishiwada, Japan; b Department of Plastic and Reconstructive Surgery, Wakayama Medical University, Wakayama, Japan; c Department of Oculoplastic Surgery, Seirei Hamamatsu General Hospital, Shizuoka, Japan

**Keywords:** Orbital fractures, Eyelids, Transconjunctival approach, Subciliary approach, Lateral canthus, Postoperative outcomes

## Abstract

**Introduction:**

Orbital floor fractures are treated using various surgical approaches; however, objective quantitative evaluation of postoperative lower eyelid morphology remains limited. This study assessed approach-specific changes in postoperative eyelid configuration.

**Methods:**

We retrospectively reviewed 35 patients with orbital floor fractures, including 26 surgically treated and 9 conservatively managed patients. Morphometric measurements were performed in all patients using standardized frontal photographs obtained ≥6 months after injury or surgery. Surgical comparisons included the transconjunctival approach (TCA; n = 7), swinging eyelid approach (SEA; n = 13), and subciliary approach (SCA; n = 6). Photographs were measured by two independent observers using ImageJ, and mean values were analyzed. Horizontal palpebral fissure width, lateral canthal height, lateral canthal angle, and lower eyelid curvature were assessed. Paired tests, Kruskal–Wallis tests, and Steel–Dwass comparisons were used, as appropriate.

**Results:**

Horizontal palpebral fissure width and lateral canthal height showed no significant intergroup differences on the affected side (χ² = 2.50, p = 0.286; χ² = 4.49, p = 0.106, respectively). Lateral canthal angle decreased in SEA and increased in SCA, with a significant SEA–SCA difference (mean difference, −7.19°; p = 0.026). Lower eyelid curvature was relatively preserved in TCA, although intergroup differences were not significant (χ² = 4.45, p = 0.108). No major complications occurred.

**Conclusions:**

TCA showed favorable preservation of lower eyelid morphology. SEA and SCA were associated with subtle, approach-specific changes in lateral canthal angle and eyelid contour. Objective morphometric assessment may complement postoperative eyelid outcome evaluation.

## Introduction

Orbital floor fractures are among the most common facial injuries and may result in diplopia, enophthalmos, and altered orbital volume or extraocular muscle dysfunction. Several surgical approaches are widely used for orbital floor fracture repair.^1^, ^2^, ^3^, ^4^

As these approaches involve the lower eyelid, postoperative malposition may adversely affect aesthetic outcomes and patient satisfaction.^5^

Among the commonly used approaches, the transconjunctival approach (TCA), swinging eyelid approach (SEA), and subciliary approach (SCA) differ in terms of surgical exposure and risk of eyelid complications. TCA avoids a cutaneous incision and is associated with favorable cosmetic outcomes.^1^^,^^5^, ^6^, ^7^ SEA incorporates lateral canthotomy with conjunctival incision, thereby allowing wide exposure while enabling careful lateral canthal reconstruction.^3^ SCA provides direct visualization but is associated with higher risk of lower eyelid malposition, visible scarring, and scleral show, although it remains frequently used in Japan.^8^

Previous studies have primarily reported qualitative aesthetic outcomes, such as visible scarring, ectropion, scleral show, and subjective cosmetic satisfaction; however, quantitative comparisons among approaches remain limited, particularly those using the unaffected side as an internal control.^9^ Therefore, objective evaluation of postoperative lower eyelid morphology is clinically important for guiding surgical approach selection and patient counseling. This study aimed to evaluate postoperative lower eyelid morphology following TCA, SEA, and SCA, and to identify approach-specific differences relevant to surgical approach selection.

## Patients and methods

This retrospective cohort study was conducted at our institution. The study protocol was approved by the Wakayama Medical University Institutional Ethics Committee (Approval No 3531), and written informed consent was obtained from all participants. The study adhered to the principles of the Declaration of Helsinki.

We retrospectively reviewed 35 patients with orbital floor fractures treated between 2015 and 2024 at our institution, including 26 patients who underwent surgical repair and 9 patients who were managed conservatively. Surgical repair was performed using TCA (n = 7), SEA (n = 13), or SCA (n = 6) (Table 1). Morphometric measurements were performed in all 35 patients, whereas comparisons among surgical approaches were performed in the 26 surgically treated patients. Conservatively managed patients were included as a reference group and were not included in comparisons among surgical approaches. Standardized frontal photographs were obtained at ≥6 months after injury or surgery to assess stable eyelid configuration. Follow-up duration was defined as the interval between injury or surgery and the date of photographic assessment. Mechanism of injury was categorized based on medical records as traffic accident, fall, assault, sports/recreational injury, or other blunt trauma. Orbital floor defect area was measured on preoperative CT images using ImageJ and included as a baseline fracture characteristic. Four parameters were measured: horizontal palpebral fissure width, lateral canthal height, lateral canthal angle, and lower eyelid curvature (Fig. 1). All standardized photographs were independently measured by two observers using ImageJ. The final value for each morphometric parameter was calculated as the mean of the two observers’ measurements. Inter-rater reliability was assessed for each parameter using the intraclass correlation coefficient (ICC) based on a two-way mixed-effects model for absolute agreement. Both observers were board-certified plastic and reconstructive surgeons with experience in periocular morphometric assessment. This method has been validated in previous studies evaluating postoperative eyelid morphology.^4^^,^^10^, ^11^, ^12^, ^13^ Surgical approaches followed established protocols for TCA, SEA, and SCA, and the use of absorbable mesh or titanium implants was consistent with prior reports.^10^^,^^14^, ^15^, ^16^

**Table 1:** Patient characteristics of surgically treated patients.

Parameter	TCA (n = 7)	SEA (n = 13)	SCA (n = 6)	Total (n = 26)
Sex (male/female)	4/3	6/7	5/1	15/11
Age (years)	38.1 ± 21.0	41.5 ± 16.0	47.8 ± 16.9	43.5 ± 20.7
Follow-up duration (months)	9.4 ± 4.7	8.8 ± 2.9	9.0 ± 3.3	9.0 ± 3.4
Orbital floor defect area (mm²)	563.2 ± 141.8	344.0 ± 95.4	511.4 ± 69.6	441.6 ± 142.9
**Mechanism of injury, n (%)**				
Traffic accident	2 (28.6)	5 (38.5)	2 (33.3)	9 (34.6)
Fall	2 (28.6)	3 (23.1)	2 (33.3)	7 (26.9)
Assault	0 (0)	2 (15.4)	1 (16.7)	3 (11.5)
Sports/recreational injury	2 (28.6)	2 (15.4)	0 (0)	4 (15.4)
Other blunt trauma	1 (14.3)	1 (7.7)	1 (16.7)	3 (11.5)

TCA, transconjunctival approach; SEA, swinging eyelid approach; SCA, subciliary approach.

**Fig. 1:**
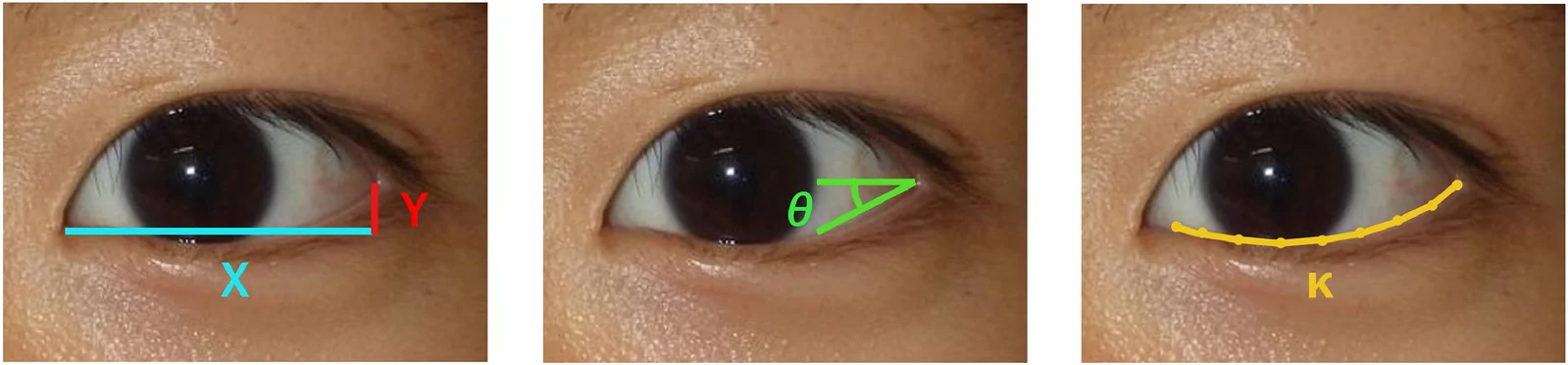
Measurement of lower eyelid morphometric parameters.

Standardized frontal photographs in primary gaze were analyzed using ImageJ (National Institutes of Health, Bethesda, MD), with the horizontal corneal diameter (11 mm) used as a reference scale. Horizontal palpebral fissure width (X) and lateral canthal height (Y) are shown on the left, lateral canthal angle (θ) in the center, and lower eyelid curvature (κ) on the right.

Statistical analyses were performed using JMP Pro version 18.2 (JMP Statistical Discovery LLC, Cary, NC, USA). Normality of the paired differences between the affected and unaffected sides was assessed using the Shapiro–Wilk test. For each morphometric parameter, comparisons between the affected and unaffected sides within each surgical approach were performed using a paired *t*-test when the paired differences were normally distributed and the Wilcoxon signed-rank test otherwise. Comparisons among the three surgical approaches on the affected side were performed using the Kruskal–Wallis test, followed by the Steel–Dwass test for pairwise comparisons. For lateral canthal angle, an additional comparison among the three groups was performed on the unaffected side to assess baseline intergroup differences. Two-sided p values < 0.05 were considered statistically significant.

## Results

The study included 35 patients with orbital floor fractures, including 26 patients who underwent surgical repair and 9 patients who were managed conservatively. Among the surgically treated patients, TCA, SEA, and SCA were performed in 7, 13, and 6 patients, respectively. Patient characteristics of the surgically treated patients according to surgical approach, including sex, age, follow-up duration, orbital floor defect area, and mechanism of injury, are summarized in Table 1. The postoperative follow-up duration among surgically treated patients was 9.0 ± 3.4 months overall, with durations of 9.4 ± 4.7 months in the TCA group, 8.8 ± 2.9 months in the SEA group, and 9.0 ± 3.3 months in the SCA group. The orbital floor defect area among surgically treated patients was 441.6 ± 142.9 mm² overall, with areas of 563.2 ± 141.8 mm² in the TCA group, 344.0 ± 95.4 mm² in the SEA group, and 511.4 ± 69.6 mm² in the SCA group.

Inter-rater reliability was good for all morphometric parameters, with ICC values of 0.838 for horizontal palpebral fissure width, 0.812 for lateral canthal height, 0.890 for lateral canthal angle, and 0.802 for lower eyelid curvature.

The comparison of lower eyelid morphometric parameters between the affected and unaffected sides for each surgical approach is shown in Table 2. Horizontal palpebral fissure width showed no significant differences between the affected and unaffected sides in the TCA (23.68 ± 1.78 vs. 23.72 ± 1.47 mm, p = 0.96), SEA (24.99 ± 2.07 vs. 25.25 ± 2.04 mm, p = 0.75), or SCA (24.18 ± 1.23 vs. 25.15 ± 1.17 mm, p = 0.19) groups. Similarly, lateral canthal height did not differ significantly between the affected and unaffected sides in the TCA (2.26 ± 1.59 vs. 1.96 ± 1.11 mm, p = 0.68), SEA (2.95 ± 0.79 vs. 3.28 ± 0.93 mm, p = 0.33), or SCA (3.70 ± 1.10 vs. 3.81 ± 1.14 mm, p = 0.68) groups.

**Table 2:** Comparison of lower eyelid morphometric parameters between the affected and unaffected sides.

Parameters		TCA	SEA	SCA	Cons.Tx
Horizontal palpebral fissure width (mm)	AS	23.68±1.78	24.99±2.07	24.18±1.23	23.32±1.1
	US	23.72±1.47	25.25±2.04	25.15±1.17	23.52±1.39
	*P*	0.96	0.75	0.19	0.74
Lateral canthal height (mm)	AS	2.26±1.59	2.95±0.79	3.70±1.10	2.7 ± 0.59
	US	1.96±1.11	3.28±0.93	3.81±1.14	2.68±0.65
	*P*	0.68	0.33	0.68	0.97
Lateral canthal angle (°)	AS	28.55±6.79	23.28±5.43	31.99±5.14	30.90±5.98
	US	28.17±5.06	27.53±6.34	27.79±4.93	32.63±5.78
	*P*	0.9	0.079	0.17	0.47
Curvature of the lower eyelid (1/mm)	AS	0.047±0.006	0.041±0.013	0.052±0.011	0.061±0.012
	US	0.045±0.010	0.045±0.012	0.044±0.012	0.058±0.008
	*P*	0.61	0.41	0.23	0.54

TCA: Transconjunctival approach; SEA: Swinging eyelid approach; SCA: Subciliary approach; Cons.Tx: Conservative treatment.

AS: Affected side; US: Unaffected side.

The lateral canthal angle was lower on the affected side than on the unaffected side in the SEA group (23.28 ± 5.43° vs. 27.53 ± 6.34°, p = 0.079), whereas it was higher on the affected side in the SCA group (31.99 ± 5.14° vs. 27.79 ± 4.93°, p = 0.17).

Kruskal–Wallis analysis demonstrated that the lateral canthal angle was the only parameter that differed significantly among the three approaches on the affected side (χ² = 7.77, p = 0.021; Table 3). No significant intergroup differences were observed in horizontal palpebral fissure width (χ² = 2.50, p = 0.286), lateral canthal height (χ² = 4.49, p = 0.106), or lower eyelid curvature (χ² = 4.45, p = 0.108). The lateral canthal angle on the unaffected side also did not differ significantly among the three approaches (χ² = 0.121, p = 0.941).

**Table 3:** Comparison of affected-side lower eyelid morphology among surgical approaches.

Parameters	χ²	df	*p*
Horizontal palpebral fissure width (mm)	2.50	2	0.286
Lateral canthal height (mm)	4.49	2	0.106
Lateral canthal angle (°)	7.77	2	0.021
Lower eyelid curvature (1/mm)	4.45	2	0.108

Steel–Dwass multiple comparisons demonstrated that the lateral canthal angle differed significantly between the SEA and SCA groups on the affected side (mean difference, −7.19°; p = 0.026), whereas no significant differences were observed between the TCA and SEA groups (p = 0.219) or between the TCA and SCA groups (p = 0.712) (Table 4). Lower eyelid curvature was closest to that of the unaffected side in the TCA group, whereas the SCA group showed a slight increase in convexity. No major complications were observed.

**Table 4:** Steel–Dwass multiple comparisons of affected-side lateral canthal angle among surgical approaches.

Comparison	Mean difference (°)	SE	Z	p value
TCA vs SEA	4.62	2.77	1.66	0.2191
TCA vs SCA	−1.70	2.17	−0.79	0.7118
SEA vs SCA	−7.19	2.78	−2.59	0.0262

TCA, transconjunctival approach; SEA, swinging eyelid approach; SCA, subciliary approach.

## Discussion

This study provides quantitative evidence suggesting that TCA favorably preserves postoperative eyelid morphology. This finding is consistent with previous reports indicating a low incidence of ectropion and scleral show following transconjunctival incisions.^1^^,^^17^ Importantly, no significant differences were detected between the unaffected and affected sides within any of the three approaches (Table 2), suggesting that gross postoperative eyelid asymmetry was limited across approaches. The lower lateral canthal angle observed in the SEA group supports prior reports suggesting that even careful lateral canthal tendon reattachment may result in subtle displacement.^3^^,^^16^ These findings extend prior qualitative observations and highlight the importance of lateral canthal configuration during surgical planning.

There was also a significant difference in affected-side lateral canthal angle between SEA and SCA, while no differences were observed among approaches on the unaffected side. These findings, combined with the results shown in Table 4, suggest that the angle changes observed were related to surgical approach rather than pre-existing asymmetry.

The SCA group showed a tendency toward widening of the lateral canthal angle and a slight increase in lower eyelid convexity. These changes are consistent with known risks of subciliary incisions, including ectropion, scleral show, and visible scarring. Recognition of these approach-specific tendencies enables more precise patient counseling and may inform the choice of surgical approach, particularly in cases where cosmetic outcomes are prioritized.^18^ Although pre- and postoperative comparison would provide the most direct assessment of surgical impact on the lateral canthal angle, trauma-related swelling can make accurate preoperative evaluation difficult, representing an inherent limitation of this study.

Overall, this study complements the existing literature by providing objective morphometric data on eyelid outcomes following three commonly used orbital floor approaches. Although qualitative assessment and patient-perceived cosmetic outcomes remain essential in clinical practice, objective morphometric analysis provides complementary information by allowing standardized and reproducible comparisons among surgical approaches. Importantly, the clinical relevance of small morphometric differences measured in millimeters or degrees remains uncertain and should be interpreted with caution. In this context, our findings suggest that TCA may be favorable when preservation of lower eyelid morphology is prioritized.^6^^,^^8^^,^^16^^,^^19^

Several limitations of this study should be acknowledged. First, the sample size was relatively small, particularly in the TCA and SCA groups, which may limit the statistical power and the generalizability of our findings. Second, our assessment relied solely on 2D photographic analysis, which may not fully capture three-dimensional contour changes. Future studies incorporating 3D imaging and volumetric analysis are warranted. Third, although all patients had at least 6 months of postoperative follow-up, the follow-up duration varied among patients, and potential long-term changes, such as delayed eyelid retraction or scar maturation, may not have been fully captured. Standardized long-term follow-up is necessary in future investigations. Fourth, subjective outcomes reported by patients, such as aesthetic satisfaction and ocular comfort, were not evaluated. Combining objective measurements with patient-reported outcome measures (PROMs) would provide a more holistic understanding of surgical success. Finally, all procedures were performed by experienced surgeons at a single institution, which may limit the generalizability of the results to other clinical settings. Multicenter studies or randomized controlled trials are needed to further validate these findings.

## Conclusion

TCA showed favorable preservation of lower eyelid morphology after orbital floor fracture repair and may be a useful approach when cosmetic outcomes are prioritized. SEA and SCA were associated with subtle, approach-specific changes in lateral canthal angle and lower eyelid contour. Recognition of these differences may assist in surgical planning and patient counseling.

## Funding

This research received no specific grant from any funding agency in the public, commercial, or not-for-profit sectors.

## Ethical approval

The study was approved by the Wakayama Medical University Institutional Review Board (Approval No 3531) and was conducted in accordance with the principles of the Declaration of Helsinki.

## Consent

Written informed consent was obtained from all participants for participation and for publication of their data and images.

## Declaration of competing interest

The authors declare no conflicts of interest.
